# Multidrug resistance-associated protein 4 is a bile transporter of *Clonorchis sinensis* simulated by *in silico* docking

**DOI:** 10.1186/s13071-017-2523-8

**Published:** 2017-11-21

**Authors:** Fuhong Dai, Won Gi Yoo, Ji-Yun Lee, Yanyan Lu, Jhang Ho Pak, Woon-Mok Sohn, Sung-Jong Hong

**Affiliations:** 1 Department of Medical Environmental Biology, Chung-Ang University College of Medicine, Seoul, 06974 South Korea; 2 Department of Convergence Medicine, University of Ulsan College of Medicine and Asan Institute for Life Sciences, Asan Medical Center, Seoul, 05505 South Korea; 3 Department of Parasitology and Institute of Health Sciences, Gyeongsang National University School of Medicine, Jinju, 52828 South Korea

**Keywords:** *Clonorchis sinensis*, Bile transporter, MRP4, ABCC4, Structure, Localization

## Abstract

**Background:**

Multidrug resistance-associated protein 4 (MRP4) is a member of the C subfamily of the ABC family of ATP-binding cassette (ABC) transporters. MRP4 regulates ATP-dependent efflux of various organic anionic substrates and bile acids out of cells. Since *Clonorchis sinensis* lives in host’s bile duct, accumulation of bile juice can be toxic to the worm’s tissues and cells. Therefore, *C. sinensis* needs bile transporters to reduce accumulation of bile acids within its body.

**Results:**

We cloned MRP4 (CsMRP4) from *C. sinensis* and obtained a cDNA encoding an open reading frame of 1469 amino acids. Phylogenetic analysis revealed that CsMRP4 belonged to the MRP/SUR/CFTR subfamily. A tertiary structure of CsMRP4 was generated by homology modeling based on multiple structures of MRP1 and P-glycoprotein. CsMRP4 had two membrane-spanning domains (MSD1 & 2) and two nucleotide-binding domains (NBD1 & 2) as common structural folds. Docking simulation with nine bile acids showed that CsMRP4 transports bile acids through the inner cavity. Moreover, it was found that CsMRP4 mRNA was more abundant in the metacercariae than in the adults. Mouse immune serum, generated against the CsMRP4-NBD1 (24.9 kDa) fragment, localized CsMRP4 mainly in mesenchymal tissues and oral and ventral suckers of the metacercariae and the adults.

**Conclusions:**

Our findings shed new light on MRPs and their homologs and provide a platform for further structural and functional investigations on the bile transporters and parasites’ survival.

**Electronic supplementary material:**

The online version of this article (10.1186/s13071-017-2523-8) contains supplementary material, which is available to authorized users.

## Background

Clonorchiasis is a major endemic disease affecting over 35 million people in Asian countries including Korea, China, Thailand and Vietnam [1–3]. *Clonorchis sinensis* infections are caused by the ingestion of raw or undercooked freshwater fish that harbor the metacercariae [4]. Complications associated with the infection increase with an increase in the intensity and duration of the infection. *Clonorchis sinensis* has also been recognized by the World Health Organization as a biological carcinogen that can induce cholangiocarcinoma in humans [5].

*C. sinensis* migrates into the bile duct of the host and lives there. However, the bile duct can be regarded as an extreme environment, since accumulation of bile juice can be toxic to the worm’s tissues and cells [6]. Among the various bile juice, lithocholic acid (LCA) has been proven to have a toxic effect on the survival of juvenile *C. sinensis* [7]. Therefore, it is important that the influx and efflux of bile acids should be balanced to prevent bile intoxication in the worm’s body. In humans, there are many importers and exporters of bile juice circulation [8], such as apical sodium-dependent bile acid transporter (ASBT), Na^+^ taurocholate co-transporting polypeptide (NTCP), Multidrug resistance protein (MRP), bile salts export pump (BSEP), and organic solute transporter (OST). Therefore, we believe that *C. sinensis* needs these bile transporters to reduce accumulation of bile acids within its body.

MRPs belong to a subfamily of the ATP binding cassette (ABC) transporter family [9, 10]. In higher animals, MRP4 is a unidirectional and distinctive bilaterally localized transporter in polarized cells, such as baso-lateral membrane of hepatocytes [11] and choroid plexus epithelial cells [12, 13]. Such tissue-specific distribution suggests that MRP4 has multiple functions. MRP4 is responsible for pumping out a broad range of substrates, including bile acids, as well as for physiological regulation via transport of cyclic nucleotides out of cells [14].

Therefore in this study, we identified and characterized *C. sinensis* MRP4 (CsMRP4), the first MRP4 in trematodes, at the *in silico*, molecular, and biochemical levels. The structure of CsMRP4 was built using homology modeling, and the structural features and bile acid-binding affinities were investigated. CsMRP4 was found to be localized mainly in the mesenchymal tissues and oral suckers of *C. sinensis* adults and metacercariae.

## Methods

### Parasites and animals

*Pungtungia herzi* (Jinju, Korea), the second intermediate host of *C. sinensis*, was ground and digested as described by Dai et al. [15]. Metacercariae were then collected from the saline-rinsed digestive leavings under a dissecting microscope. Next, New Zealand white rabbits (2.3 kg; Koatech, Seoul, Korea) were infected with 200 metacercariae per rabbit twice in 1 week. Adult *C. sinensis* were then recovered from the rabbit livers after 2 months and stored in a  -80 °C freezer until use. Female 7 week-old BALB/c mice (Orient Bio Inc., Gyeonggi-do, Korea) were immunized with a bacterially-produced recombinant protein.

### CsMRP4 cDNA

A putative MRP4 polypeptide sequence (GenBank ID: GAA49862.1) of *C. sinensis* was retrieved from the National Center for Biotechnology Information (NCBI) database. The coding DNA sequence (CDS) was obtained from the *C. sinensis* DNA scaffold (GenBank ID: DF142991.1) to which it belonged. DNA-walking was performed twice for CsMRP4-I and CsMRP4-II due to the long size (approximately 4 kb) of the products. Two sets of PCR primers were designed according to CDS and synthesized (Bioneer, Daejeon, Korea) (Additional file 1: Table S1). Total cDNA of *C. sinensis* was prepared as described previously [15], and 50 ng per reaction was used as the template for DNA-walking. PCR amplification was performed under the following conditions: pre-denaturation (94 °C for 5 min), amplification phase with 35 cycles (94 °C for 30 s, 55 °C for 30 s, 72 °C for 2 min 15 s), and final extension (72 °C for 10 min). The PCR products were then purified using the QIAquick PCR purification kit (Qiagen, Hilden, Germany) and sequenced (Macrogen Inc., Seoul, Korea). The CsMRP4-I and CsMRP4-II sequences were used for assembling the putative CDS and translated into amino acid (aa) sequences. However, CsMRP4 was assumed to be incomplete at the 5′-end upon comparison with MRP4 of other species. Therefore, 5′-rapid amplification of the cDNA ends (5′-RACE) was carried out to obtain the entire CDS. Total cDNA of *C. sinensis* was synthesized using the SMARTer™ RACE cDNA amplification kit (Clontech, Mountain View, CA, USA) according to the manufacturer’s instructions. The missing 5′-end of CsMRP4 was amplified by RACE-PCR run using the 5′-RACE universal primer mix (UPM) and gene specific reverse primer (GSP). The PCR product was then confirmed using nested PCR, purified, and subjected to TOPO TA cloning (Invitrogen, Carlsbad, CA, USA). Through blue-white screening, the positive white colony was selected and reconfirmed by rapid colony PCR. Its plasmid DNA was extracted using the Plasmid Miniprep kit (Qiagen, Seoul, Korea) and sequenced (Macrogen Inc., Seoul, Korea). The primers used for RACE-PCR, DNA-walking, and multiple sequencing are listed in Additional file 1: Table S1.

### *In silico* methods for characterizing sequence features

For CsMRP4, the isoelectric point (pI) and molecular weight (Mr) was estimated using the ExPASy ProtParam Tool (http://web.expasy.org/protparam/). CsMRP4 was blasted against UniProtKB/Swiss-Prot v. 2017_07 [16]. Domain organization and residue annotation were conducted using the Conserved Domain Database (CDD) [17] and InterProScan v. 64 [18].

### Phylogenetic analysis

In order to confirm that CsMRP4 belongs to a MRP subfamily and to infer its phylogenetic relationship with the ABCC and ABCB subfamilies, 12 canonical ABCC proteins and 11 canonical ABCB proteins were retrieved from UniProtKB/Swiss-Prot v. 2017_07 [16]. Multiple sequence alignment was performed using the L-INS-i method of MAFFT v. 7.299 [19]. An evolutionary history was inferred by employing the neighbor-joining (NJ) method using MEGA v. 6.06 [20]. All the positions containing gaps and missing data were eliminated.

### Homology modeling and refinement

The standard protocol of YASARA Structure v. 17.6.5 [21] was used to build the homology models of CsMRP4. To obtain these models, PSI-BLAST [22] was carried out against PDB entries (updated August, 2017) [23]. After building the homology models for each template, the models were submitted to high-resolution energy minimization using a YASARA force field [24]. The result was then validated to ensure that the refinement did not move the model in the wrong direction. Finally, a hybrid homology model was obtained by combining the best scoring parts of the four models. In addition, potential errors in the 3D models were evaluated using a Ramachandran plot [25] and ERRAT [26].

### Structure-based function analysis

Structural conservation was calculated and visualized using ENDscript/ESPript v. 3.0 [27] with the PDBAA95 database, E-value of 1e-12, and contact range of 2.7 Å. COACH [28] was used to predict ligand-binding sites in CsMRP4. SDF files for bile acids were retrieved from the PubChem database [29] as of August 2017 and transformed into the MOL2 format using OpenBabel [30]. Bile acids were docked into CsMRP4 using PyRx v. 0.8 [31], which includes AutoGrid [32] and AutoDock Vina [33]. A grid box extended to all membrane-spanning domains (MSDs) of CsMRP4; no information regarding the exact location of the binding sites of the various bile acids was available. Active site dimensions were set as the grid size of center X: 25.6 Å, center Y: -13.5 Å, and center Z: -6.5 Å, and 8 maximum exhaustiveness was calculated for each bile acid. All structure visualizations were carried out using UCSF CHIMERA v. 1.10.2 [34] and PyMOL Molecular Graphics System v. 1.7.4.5 (Schrödinger, LLC., New York, NY, USA).

### Quantitative measurement of CsMRP4 developmental expression

To evaluate the mRNA expression level in different developmental stages, quantitative real time PCR (Q-rt.-PCR) was performed. Primers were designed using Oligo-primer analysis software v. 6.71 (Molecular Biology Insights, Cascade, WA, USA) (Additional file 1: Table S1). *Calcyphosine* (*CAP*) and *phosphoglycerate kinase* (*PGK*) were employed as the reference genes [35]. Q-rt.-PCR reaction mixtures were prepared in triplicate using LightCycler FastStart DNA Master SYBR Green I Kit (Roche, Mannheim, Germany), with each reaction containing 50 ng of total cDNA of the adults or metacercariae. Q-rt.-PCR was performed on LightCycler 2.0 (Roche, Penzberg, Germany) with the following thermal cycle parameters: pre-heating (95 °C for 15 min), 40 cycles of 95 °C for 10 s, 48 °C for 10 s, and 72 °C for 30 s. The relative transcription ratio was calculated according to the 2^-ΔΔCt^ method [36].

### Production of recombinant protein

A cDNA fragment encoding CsMRP4-NBD1 was amplified using PCR (Additional file 1: Table S1). The purified PCR product was then subcloned into pET23b and confirmed by colony PCR and restriction enzyme digestion. Plasmid DNA of the positive clone was extracted using the QIAprep Spin Miniprep kit (Qiagen, Hilden, Germany) and sequenced (Macrogen, Seoul, Korea). The correct construct was then transformed in *Escherichia coli* BL21[DE3]pLysS (Novagen, San Diego, CA, USA) by heat-shock at 42 °C for 30 s and spread on LB/ampicillin/chloramphenicol agar. After overnight incubation at 37 °C, a single colony was picked from the LB plate and grown in LB/ampicillin liquid medium by shaking vigorously at 37 °C. The recombinant(r) CsMRP4-NBD1 was then induced with 0.1 mM isopropyl-β-D-thiogalactopyranoside (IPTG) (TaKaRa, Shiga, Japan) for 5 h. The bacteria were then harvested, and recombinant protein was purified as described previously [15].

### Production of mouse immune serum

The rCsMRP4-NBD1 was separated by 12% sodium dodecyl sulfate polyacrylamide gel electrophoresis (SDS-PAGE) and cut off alone to obtain specific antigens for mouse immunization. The gel slice was equilibrated and homogenized in pre-cooled 1× PBS by complete grinding. The liquid homogenate containing rCsMRP4-NBD1 was then injected into BALB/c mice according to an immunization method [37]. Blood was drawn from the eye and stored at room temperature for 1 to 2 h. The immune serum was obtained by centrifugation at 4000× *rpm* for 20 min. In order to examine the antibody titer in the immune serum against rCsMRP4-NBD1 and native CsMRP4, *C. sinensis* crude extracts were prepared using the Mem-PER Plus Membrane Protein Extraction Kit (Thermo scientific, Rockford, USA) following the manufacturer’s instructions. The crude antigen was then examined to determine its concentration using the Bio-Rad protein assay (Bio-Rad, Hercules, CA, USA) and then stored as aliquots at -70 °C until use.

The reactivity of the antibody was checked by western blot against recombinant protein and immuno-enhanced chemiluminescence (ECL) against native CsMRP4 in crude extracts. The rCsMRP4-NBD1 and *C. sinensis* crude antigens were loaded onto SDS gels for electrophoresis and then transferred to nitrocellulose membranes (GE Healthcare Life Sciences, Seoul, Korea). The membranes were then blocked using 5% skim milk in PBS/0.05% Tween20, followed by incubation with mouse immune serum at 1:400 at 4 °C overnight and then with goat-anti-mouse-IgG alkaline phosphatase-conjugated antibody (Sigma-Aldrich, St. Louis, MO, USA) at 1:5000 for western blotting or with peroxidase-conjugated AffiniPure Goat Anti-Mouse IgG antibody (Jackson ImmunoResearch Inc., West Grove, PA, USA) at 1:10,000 for ECL at room temperature for 2 h. Normal mouse serum was used as the negative control. The recombinant protein was visualized by color developing in BCIP/NBT (Sigma-Aldrich, St. Louis, MO, USA). The native CsMRP4 was detected using a ECL solution kit (Bio Sesang, Seoul, Korea) and visualized using ImageQuant LAS 4000 (GE Healthcare Bio-Sciences, Amersham, UK).

### Immunohistochemical staining

Paraffin block preparation and immunohistochemical staining were performed using our previously described methods [15]. Mouse anti-NBD1 immune serum diluted at 1:200 served as the primary antibody. Normal mouse serum was used as the negative control. Dako EnVision + System-horseradish peroxidase-labeled polymer anti-mouse IgG (Dako Cytomation, Glostrup, Denmark) diluted at 1:400 was used as the secondary antibody.

## Results and discussion

### Identification and molecular characteristics

The complete coding cDNA sequence (4410 nt) of *CsMRP4* was obtained through DNA-walking and 5′-RACE on the *C. sinensis* total cDNA. Its open reading frame was 1469 aa in length (Additional file 2: Figure S1 and Additional file 3: Figure S2). The Mr. of CsMRP4 was about 165.5 kDa and its pI was estimated to be 6.5. BLASTP was performed against the UniProtKB/Swiss-Prot database [16], which is a high-quality, manually annotated, and non-redundant protein database. Annotation information from NCBI non-redundant and UniProtKB/TrEMBL databases need to be further reviewed, since those contents were generated using *in silico* annotation or large-scale functional prediction. CsMRP4 is the closest to human MRP4 (HsMRP4) (UniProt ID: O15439) with an E-value of 5.3e-147 and identity of 44.2%, followed by HsMRP6 (UniProt ID: O95255) of 8.8e-89 and 38.4%, *Mus musculus* MRP3 (MmMRP3) (UniProt ID: B2RX12) of 5.1e-104 and 38.2%, and MmMRP5 (UniProt ID: Q9R1X5) of 2.9e-115 and 38.0%. CsMRP4 was significantly matched to multiple MRPs since the highest-scoring pairwise alignment was found predominantly in the NBD2 region, which is particularly conserved among ABC family transporters [38]. As a subfamily of the ABC family of transporters, the MRP subfamily contains 12 members including MRP1-9, cystic fibrosis transmembrane conductance regulator (CFTR), sulfonylurea receptor 1 (SUR1), and SUR2 [9, 10]. The highest identity (39.0%) was observed between CsMRP4 and HsMRP4 in comparison with canonical human MRP/SUR/CFTR subfamily (Additional file 4: Table S2). Thus, this clone was designated as CsMRP4.

### MRP-specific functional domains

Functional domains in CsMRP4 revealed diverse characteristics of ABC transporters. There were “ABC transporter type 1, transmembrane domain” (InterProScan ID: IPR011527) in two regions, aa64–378 and aa831–1162. Two domains, MSD1 and MSD2, consisted of six transmembrane α-helices each. As intracellular NBDs that linked with the MSDs, “ABCC_MRP_domain1” (CDD ID: cd03250) for NBD1 and “ABCC_MRP_domain2” (CDD ID: cd03244) for NBD2 were found in the aa407–603 and aa1174–1394 regions, respectively. CsMRP4 had a single four-domain organization of MSD1-NBD1-MSD2-NBD2, which is common to all short forms of the ABCC subfamily, such as MRP4, 5, 8, 9, and CFTR [14].

Like in typical MRPs, there were several conserved motifs in the NBD1 of CsMRP4, such as an ATP-binding site (^441^GCxKSSx_26_Qx_78_DDx_31_N^585^), ABC transporter signature motif (^528^LSGGQKARIG^537^), Walker A/P-loop (^438^GPVGCGKS^445^), Walker B (^548^FLLLDD^553^), D-loop (^556^AAVD^559^), Q-loop/lid (^470^YMPQ^473^), and H-loop/switch region (^581^LLVTNQL^587^). These motifs play pivotal roles in transporting substrates via conformational changes between outward-facing and inward-facing forms. Dimerization of two NBDs forms the nucleotide-binding site between the Walker A/P-loop, Walker B, and ABC signature motif. The bound ATP is hydrolyzed to provide energy in order to efflux endogenous and xenobiotic substrates from cells to the extracellular milieu [39].

### Phylogenetic inference

**Fig. 1:**
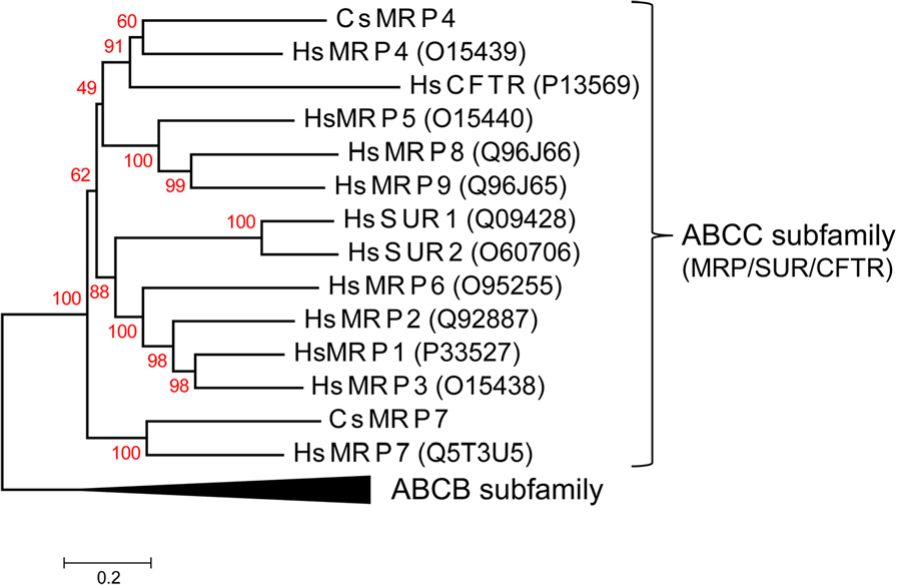
Phylogenetic relationship between CsMRP4 and members of the MRP/SUR/CFTR subfamily. Bootstrap values (1000 replicates) are shown next to the branches. Scale bar represents amino acid substitutions. UniProtKB/Swiss-Prot IDs for the ABCC subfamily are shown in parentheses. IDs for the ABCB subfamily are P-gp1 (P08183), ATP1 (Q03518), ATP2 (Q03519), P-gp3 (P21439), ABCB5 (Q2M3G0), MT-ABC3 (Q9NP58), ABC7 (O75027), M-ABC1 (Q9NUT2), TAPL (Q9NP78), M-ABC2 (Q9NRK6), and BSEP (O95342)

An NJ method-based phylogenetic inference verified that CsMRP4 belongs to the MRP/SUR/CFTR subfamily by comparing it with the 12 members of subfamily C and 11 members of subfamily B of the ABC family (Fig. 1). Members of the ABCB subfamily were clearly out-grouped as one cluster despite the close similarity in terms of both sequence and structure between MRP and P-glycoprotein (P-gp) of ABCB members [39]. Moreover, CsMRP7 (GenBank ID: AOE23877.1), which was annotated using the same approach, was grouped with HsMRP7.

### 3D homology model of CsMRP4

**Fig. 2:**
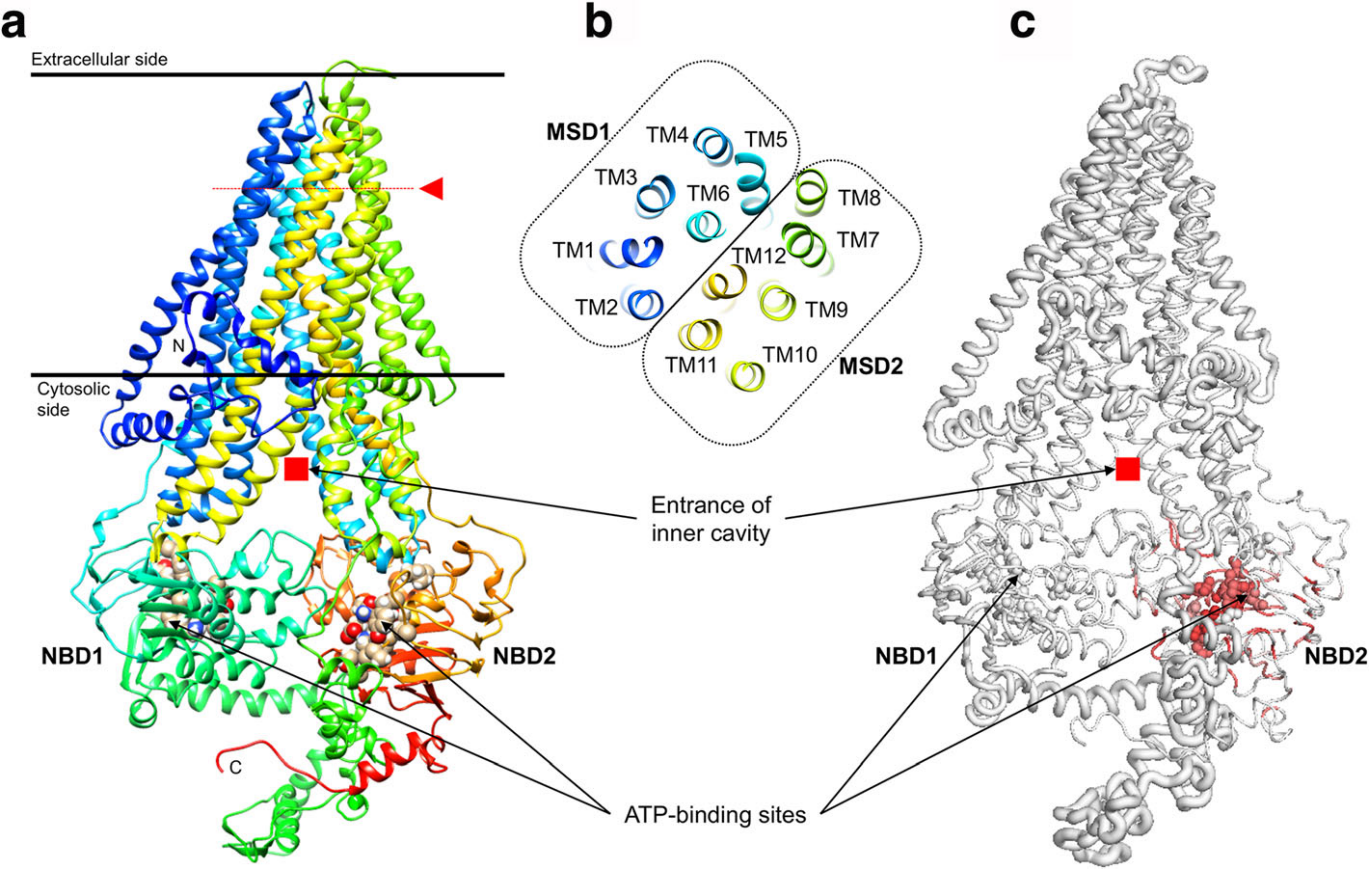
Structural characterization and conservation of CsMRP4. **a** A 3D homology model was built based on the solved structure templates. The α-helix and β-strand are depicted as ribbon diagrams, and coiled-coil is depicted as a line. TM α-helices are colored and numbered from the N-terminus (*blue*) to the C-terminus (*red*). The *red rectangles* indicate the entrance of inner cavity, and spheres correspond to residues coordinating ATP-binding sites. **b** TM α-helices, as viewed perpendicular to the horizontal plane marked with *red arrowheads*. **c** The degree of sequence conservation is colored using a color gradient from *white* (divergent) to *red* (conserved). Structural conservation corresponds to the radii of the backbone sausage representation, which is proportional to the root-mean-squared deviation at each position between structure alignments. PDB IDs of the identified homologs are as follows: 5W81_A, 5UAK_A, 5UJ9_A, 4C3Z_A, 2PZG_A, 3GD7_A, 4Q4J_B, 1R0Z_A, 2HYD_A, 4Q7M_B, 4Q4J_A, 5DGX_A, 5IDV_A, 4MYC_A, 3WMF_A, 5MKK_A, 1MV5_A, 4MRN_A, 3NH6_A, 2FFB_A, 5EUM_A, 4F4C_A, 2GHI_A, 5MKK_B, 4Q9H_A, 4AYW_A, 3VX4_A, 5U1D_B, 5U1D_A, 4PL0_A, 5L22_B, 4U00_A, 4K8O_A, 4MKI_B, 4HUQ_B, 3TUJ_C, 5NIK_J, and 5JSZ_A. Structural alignment and image rendering were carried out using ENDscript and PyMOL (See details in the Methods section)

Several MRP4-related PDB structures were found from the PSI-BLAST search with experimentally characterized PDB structures, although there is no solved PDB structure of MRP4 elucidated so far. YASARA Structure selected the templates, such as *Bos taurus* MRP1 (PDB ID: 5UJA) with 35.2% identity, *Caenorhabditis elegans* P-gp (PDB ID: 4F4C) with 19.1% identity, MmMRP1 (PDB ID: 4M1M) with 18.9% identity, and MmMRP1 (PDB ID: 4Q9H) with 18.3% identity. Each homology model, built with each template, was refined by unrestrained high-resolution energy minimization using the latest knowledge-based YASARA force field [24]. As a final model, a hybrid homology model was assembled by combining the best scoring parts of the four models and then refined with energy minimization. Out of 1469 residues, 1425 residues were modeled, omitting 44 C-terminal residues, since YASARA Structure does not perform ab initio or threading modeling (Fig. 2).

The final model proved highly accurate based on the following validation. The overall Z-score of the resulting hybrid model was -1.4 using internal quality evaluation of YASARA Structure. A Z-score indicates the number of standard deviations the model quality is away from the average high-resolution X-ray structure. Moreover, a Ramachandran plot [40] of the final model showed that 90.7% of all the residues were found in the most favored regions, 8.4% in additional allowed regions, and only 0.3% in disallowed regions (Additional file 5: Figure S3). These results indicated that the backbone dihedral angles were highly accurate. The ERRAT value, as an overall quality score, was 98.4% (Additional file 6: Figure S4). The final model of the CsMRP4 in PDB format can be found in Additional file 7.

**Fig. 3:**
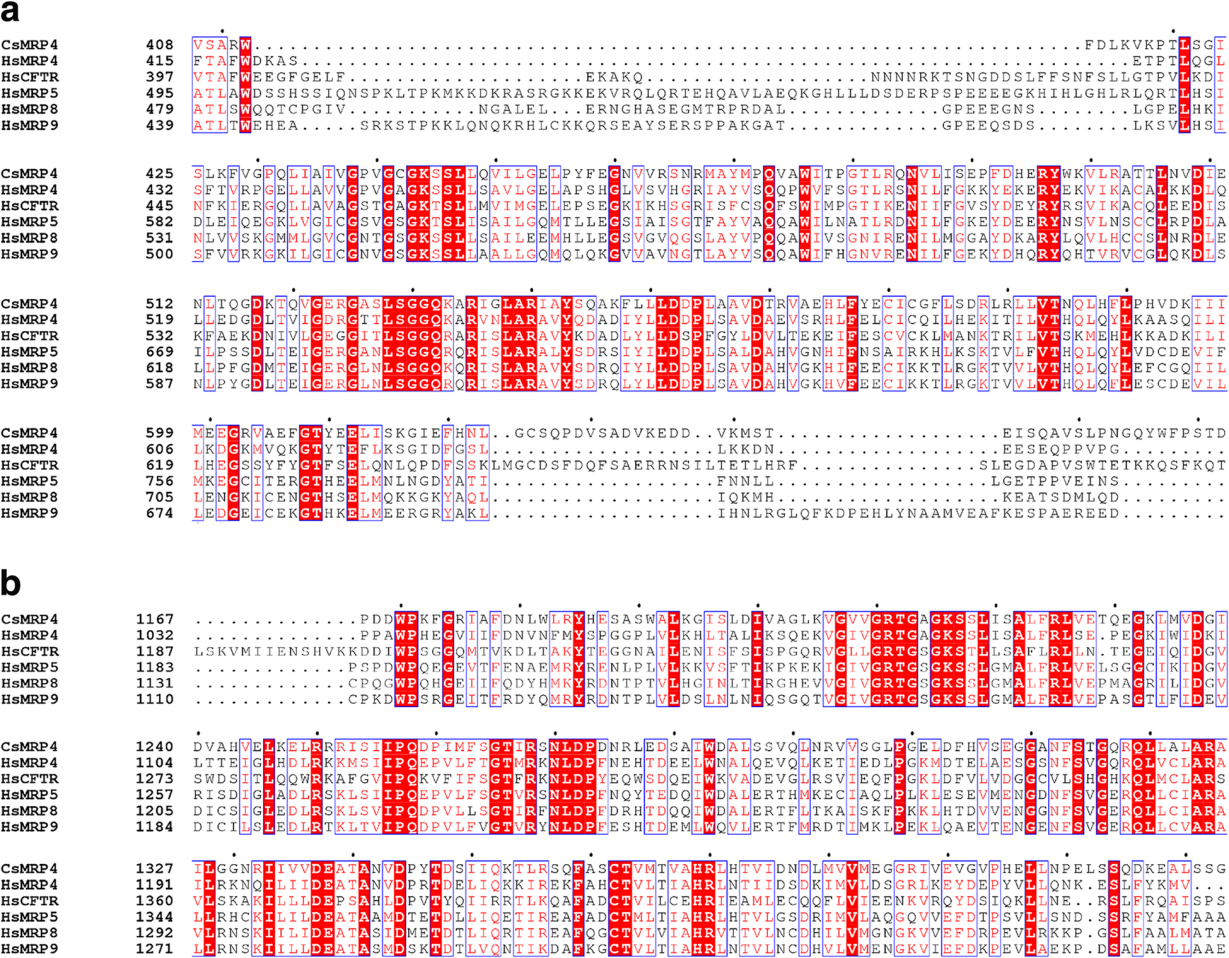
Comparison of the amino acid sequence of CsMRP4 with short forms of the ABCC subfamily. CsMRP4 was aligned with HsMRP4, 5, 8, 9, and CFTR using MAFFT and rendered using ESPript. Out of the alignment profile, the NBD1 region (**a**) and NBD2 region (**b**) were selected for visualization. *Red* bold and *red* letters indicate identical and similar amino acid residues, respectively. Conserved sequences are indicated by a box if more than 70% of the residues are similar

The 3D structure formed MSD1-NBD1-MSD2-NBD2 as a common structural fold of ABC transporters (Fig. 2a). MSD1 and MSD2 were made up of TM1–6 and TM7–12, respectively (Fig. 2b). When CsMRP4 was compared structurally with the 38 homologs from PDB entries using ENDscript [27] with strict parameters, two NBDs were found to be highly conserved and two MSDs were less conserved (Fig. 2c). Furthermore, NBD2 of CsMRP4 was significantly more conserved than NBD1. At the sequence level, even though CsMRP4 was compared with five short forms of the ABCC subfamily, NBD2 contained 56 identical residues but NBD1 had only 41 identical residues (Fig. 3a, b). These results corroborate previous findings showing that NBD2 of CsMRP7 is more conserved than NBD1 at the structural level [15].

### Docking simulation for bile acid binding

Various substrates were moved out via binding with MRP4 transporters. The export of inhibitors and bile acids can confer drug resistance and bile recirculation, respectively. Three MRP1 proteins and a P-gp, used as the four PDB templates, showed an open inward-facing conformation with inner cavity, which appears to be suitable for substrate uptake. These templates provided a possible structural foundation to perform *in silico* prediction of ligand binding by docking simulation. Thus, we investigated ligands and their binding sites for CsMRP4 using two methods. First, probable ligands were analyzed based on the identification of analogs with similar binding sites as the solved structures using COACH [28]. Then, Mg^2+^ and ATP were predicted to bind to NBD1 and NBD2 of CsMRP4 based on data for HsMRP1 [41] (Table 1 and Fig. 2a). Mg^2+^ is necessary for ATP hydrolysis and results in the formation of Mg-ATP dimers [42]. Moreover, the cyclic peptide inhibitor, QZ59-SSS (a.k.a. OZ-VAL or 2 J8), was predicted to bind to CsMRP4 according to the ligand-bound pockets of three experimentally characterized P-glycoproteins [43–45] (Table 1). Among them, Ile at the position 1093 was commonly involved in coordinating the inhibitor.

**Table 1:** Ligand and ligand-binding residues predicted using COACH

Ligand	Region	Consensus binding residues	PDB template
ATP	NBD1	W412, T420, V440, G441, C442, G443, K444, S445, S446, Q473	2CBZ_A
Mg^2+^	NBD1	S445, Q473	2CBZ_A
AMP-PNP^a^	NBD2	Y1185, A1192, T1212, G1213, A1214, G1215, K1216, S1217, S1218, V1227, Q1258, E1338, H1369	2ONJ_A
Mg^2+^	NBD2	S1217, Q1258, D1337, E1338, V1367	4FWI_B
2J8	Cavity	L94, P98, M101, S348, L864, I1093, V1097	4M2T_A
QZ59-VAL	Cavity	P98, I1093	4Q9J_A
0JZ	Cavity	Y349, L353, I1093, I1118, V1122	3G61_B

^a^AMP-PNP is an ATP analogue [44]

**Fig. 4:**
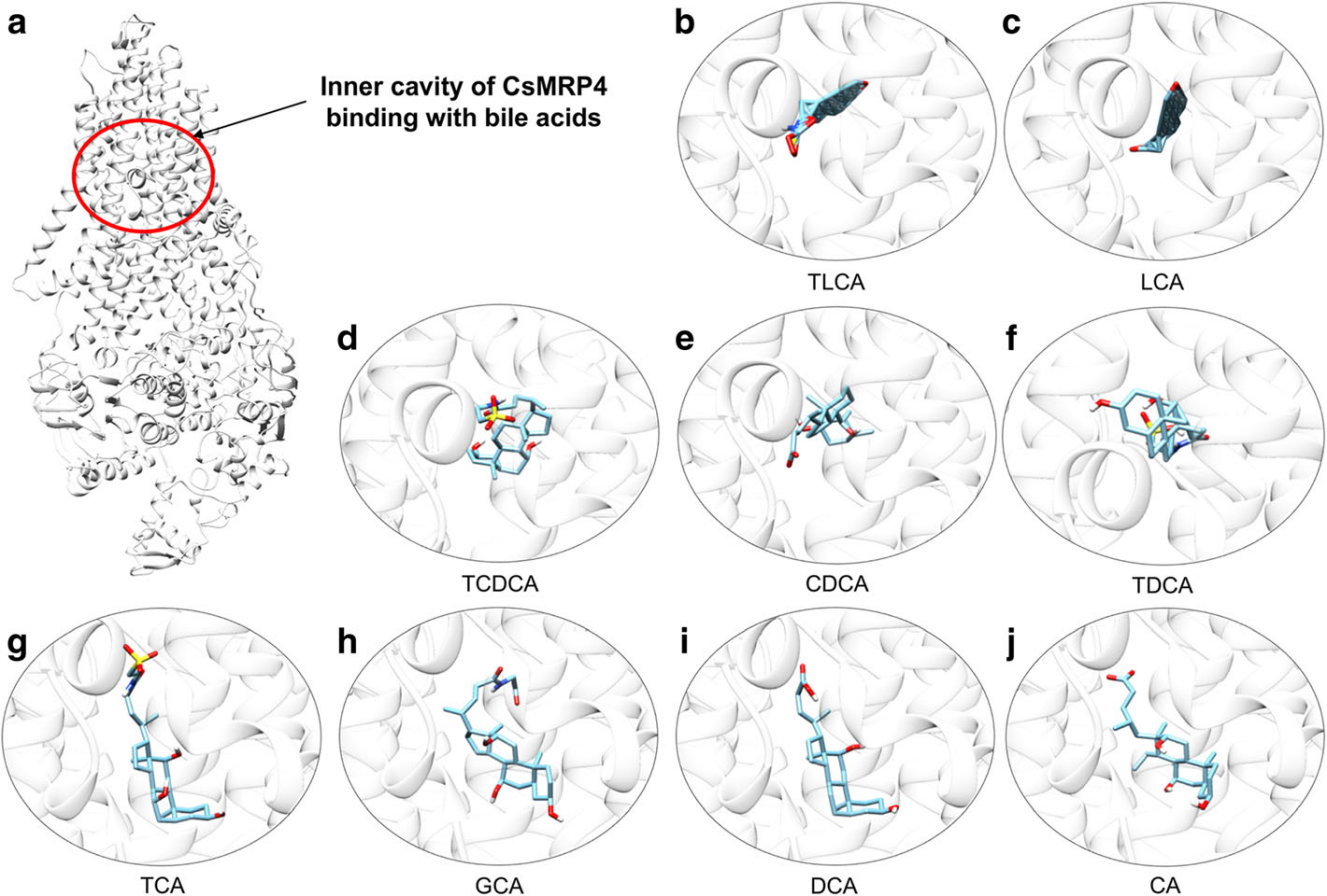
Best docking conformation of bile acids with CsMRP4. The 3D model of CsMRP4 is visualized as a *white* ribbon diagram, which is depicted in transparency (90%) to show the bile acid docked deep into the inner cavity (**a**). The *red circle* indicates the inner cavity for bile acid binding. The results are arranged according to the order of calculated binding affinities of complex bile acid-CsMRP4, as listed in Table 2: **b** TLCA, **c** LCA, **d** TCDCA, **e** CDCA, **f** TDCA, **g** TCA, **h** GCA, **i** DCA, **j** CA

Secondly, docking simulations were performed using AutoDock Vina [33] to evaluate the binding energies of CsMRP4 with nine bile acids (Table 2 and Fig. 4). All the bile acids tested bound favorably to the inner cavity of CsMRP4 (Fig. 4a). Taurolithocholic acid (TLCA) (Fig. 4b) and LCA (Fig. 4c) showed the highest affinities with CsMRP4, whereas deoxycholic acid (DCA) (Fig. 4i) and cholic acid (CA) (Fig. 4j) revealed moderate affinities. Interestingly, our docking results are in line with previous transport assay data, which indicated that TLCA bound favorably to MRP4 at a low concentration, but other bile acids needed much higher concentrations [46]. TLCA had the highest affinity for MRP4 overexpressed in HEK cells, followed by taurochenodeoxycholic acid (TCDCA), taurodeoxycholic acid (TDCA), taurocholic acid (TCA), glycocholic acid (GCA) and cholic acid (CA). We also then added more primary and secondary bile acids such as LCA, chenodeoxycholic acid (CDCA), and DCA. Among them, LCA at 2–4 μM concentration was reported to have a significant adverse effect on the survival of juvenile *C. sinensis* [7]*.* Thus, the high affinity of LCA could be required for removing LCA from the worm’s body for survival. However, these findings remain to be established at the biochemical level, which needs to be studied in the future.

**Table 2:** Docking results between CsMRP4 and bile acids using AutoDock Vina

Bile acids	PubChem ID	Binding energy (kcal/mol)	No. of configurations
Taurolithocholic acid (TLCA)^a^	SID 103579026	-13.4	3
Lithocholic acid (LCA)	SID 103542513	-12.2	3
Taurochenodeoxycholic acid (TCDCA)^a^	SID 312642451	-10.1	5
Chenodeoxycholic acid (CDCA)	SID 24875071	-9.9	7
Taurodeoxycholic acid (TDCA)^a^	CID 2733768	-9.7	5
Taurocholic acid (TCA)^a^	SID 828139	-9.3	6
Glycocholic acid (GCA)^a^	SID 177011773	-9.2	7
Deoxycholic acid (DCA)	CID 222528	-8.4	5
Cholic acid (CA)^a^	SID 223730521	-8.1	5

^a^Binding affinities of bile acids to MRP4 in HEK cells [46]

### Developmental expression and tissue distribution

**Fig. 5:**
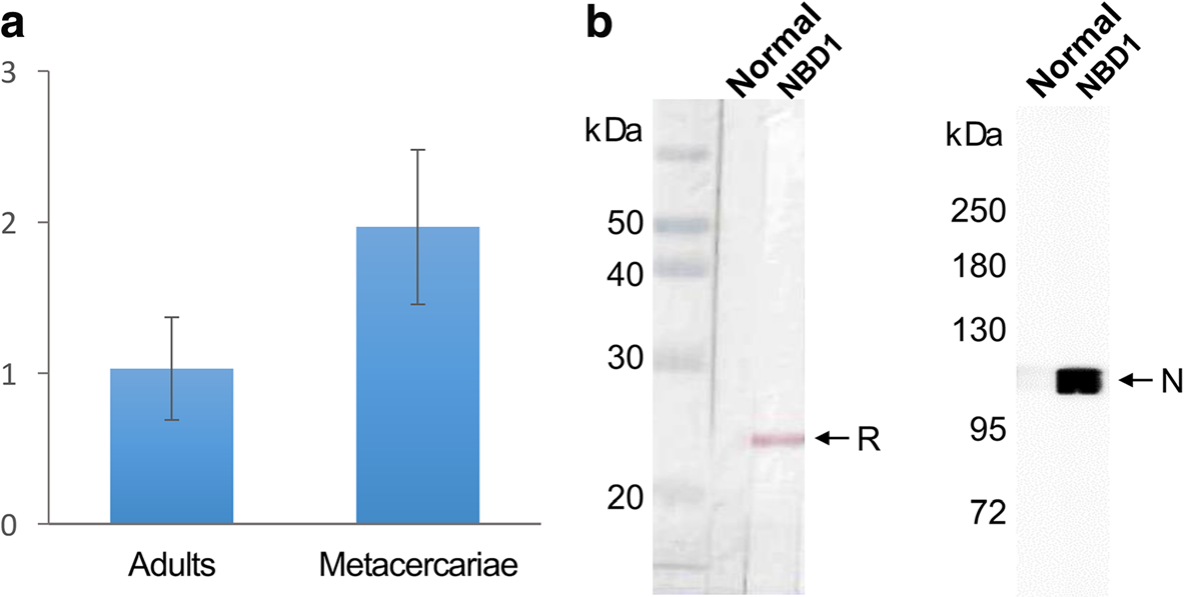
Differential expression of the CsMRP4 and reactivity of mouse anti-NBD1 immune serum. **a** Relative mRNA level of *CsMRP4* gene in the adults and metacercariae of *C. sinensis*, measured using Q-rt.-PCR. **b** Reaction detected by western blot and immuno-ECL. The mouse immune serum reacted well with rCsMRP4-NBD1 (left) and specifically detected native CsMRP4 (right) in the crude extracts of *C. sinensis*. Lane NBD1: mouse anti-NBD1 immune serum; Lane Normal: normal mouse serum; *Abbreviations*: R, recombinant protein; N, native CsMRP4

CsMRP4 mRNA was expressed at both developmental stages, in the metacercariae and in the adults, but the expression in the metacercariae was 1.91 times higher (Fig. 5a). This result suggested that the metacercariae might need the transcript for the efflux of bile acids from the fluke’s body during their survival in the bile duct of the final host. In the metacercarial stage of *C. sinensis*, diverse genes have been reported to be highly expressed in response to environmentally induced changes, such as sodium/bile acid cotransporter and several heat-shock proteins [47]. Recently, the mRNA level of *CsMRP7* was also reported to be elevated in the metacercariae [15].

**Fig. 6:**
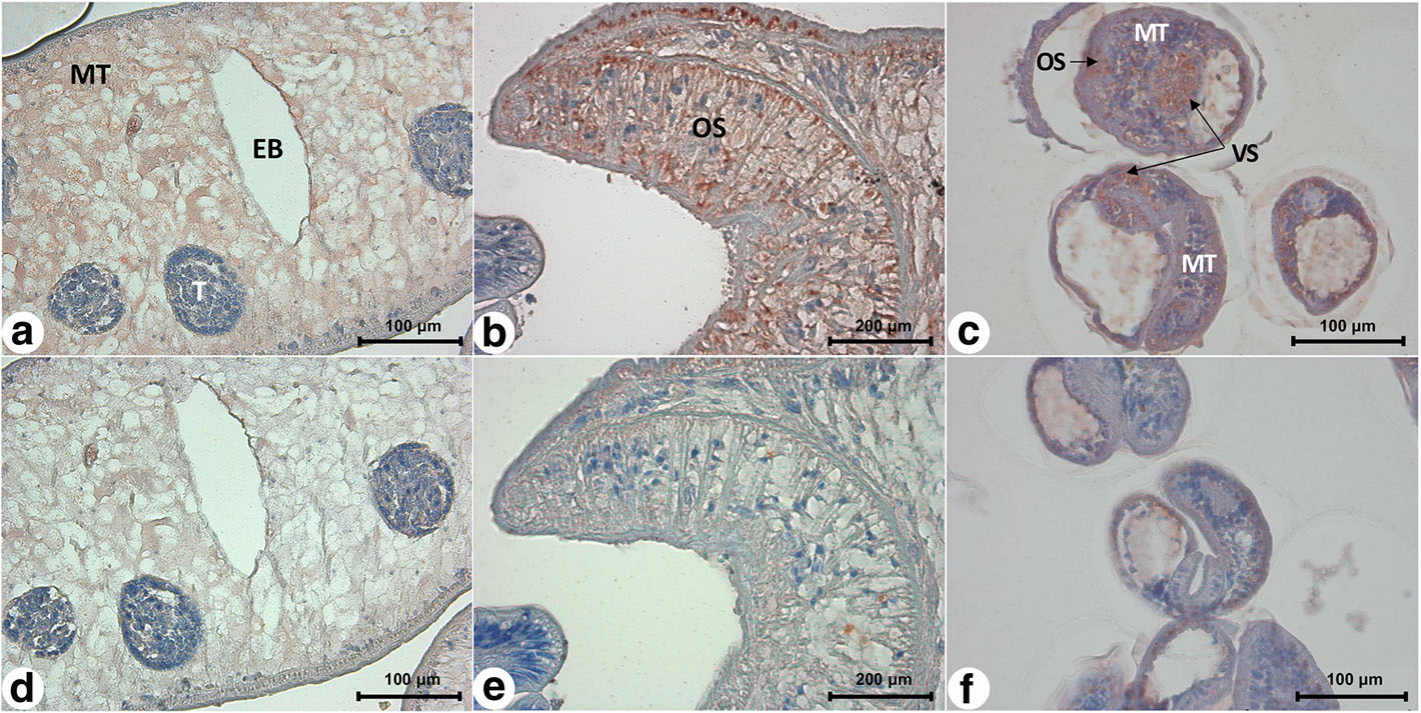
Localization of CsMRP4 in *C. sinensis* adults (**a**, **b**, **d**, **e**) and metacercariae (**c**, **f**) detected by immunohistochemistry. Top panels (**a** **-** **c**) were stained with mouse anti-NBD1 immune serum and bottom panels (**d** **-** **f**) were stained with normal mouse serum. *Abbreviations*: EB, excretory bladder; MT, mesenchymal tissue; OS, oral sucker; T, testis; VS, ventral sucker

The tissue distribution of CsMRP4 in both adults and metacercariae was investigated via immunohistochemistry. The NBD1 region was chosen as the immune antigen for mice immunization, since it was more specific than NBD2 and the non-membrane-spanning region. With this strategy, we successfully produced and purified NBD1 of ABCC subfamily transporters [15]. The 6× histidine-tagged rCsMRP4-NBD1 was then purified using Ni-NTA agarose (Additional file 8: Figure S5). The mouse anti-CsMRP4-NBD1 immune serum reacted well with rCsMRP4-NBD1 (24.9 kDa) by apparently detecting the native CsMRP4 in the crude extract of the adult worm (Fig. 5b). It was, therefore, applied to immunohistochemical staining. CsMRP4 was distributed mainly in the oral sucker and mesenchymal tissues of the adults (Fig. 6a, b) and metacercariae (Fig. 6c). Moreover, the ventral sucker of the metacercariae showed strong localization of CsMRP4.

CsMRP4 was mainly localized in the mesenchymal tissues in our study. In trematodes, several transporters have been found to be expressed in mesenchymal tissues. BSEP and MRP1 of adult *Fasciola hepatica* have been shown to be localized in not only mesenchymal tissues but also the tegumental cell layer, implying that the two transporters facilitate the diffusion of bile salts and chemicals in flukes [48]. CsMRP7, which might be involved in drug resistance, has also been found to be expressed in the mesenchymal tissues [15]. It is therefore speculated that mesenchymal tissues may be regions of strategic importance for transporter functions throughout the body of flukes.

The function of CsMRP4 seems to be to export bile acids from the worm’s body, which is similar to the function of typical MRP4. *C. sinensis* adults and metacercariae immerse themselves in bile juice, which can exert toxic effects and impair the tissues and cells of the worm’ bodies. Bile acids have also been reported to decrease the locomotive cycles of juvenile *F. hepatica* and to provoke parasite death [49]. Together, these data suggest that *C. sinensis* needs to dilute the high concentrations of bile acids in the interior of the body by pumping them out and that CsMRP4 plays a role in transporting bile acids in coordination with other bile acid exporters.

## Conclusions

In summary, we cloned and characterized CsMRP4 using computational, molecular, and biochemical approaches in this study. In addition to structural similarities, sequence similarities were also found between CsMRP4 and human MRP4 (39% identity), and CsMRP4 was confirmed as belonging to the ABCC family. A reliable tertiary structure of CsMRP4 was also modeled and shown to have a common structural fold, MSD1-NBD1-MSD2-NBD2. When binding affinities of CsMRP4 with nine bile acids were tested through virtual docking simulation, the results indicated that CsMRP4 could be regarded as a bile transporter. The NBD2 of CsMRP4 was conserved more than NBD1, which was therefore used as a CsMRP4-specific antigen for subsequent immunohistochemistry experiments. In the metacercariae and adults of *C. sinensis*, CsMRP4 was found to be mainly distributed in mesenchymal tissues, which suggested that these tissues are regions of strategic importance for transporter functions throughout the fluke’s body. These findings suggest that CsMRP4 plays a role in exporting bile acids and inhibitors. The results from this study will also serve as a platform for further research on other bile transporters and homologues in flukes.

## Additional files

Additional file 1: Table S1. Primer sets used to amplify CsMRP4 cDNA fragments by PCR. (PDF 20 kb)

Additional file 2: Figure S1. Strategy for obtaining the entire coding cDNA sequence of the *CsMRP4* gene. a Whole cDNA (4410 bp) was confirmed by combining 5′-RACE PCR and DNA-walking. The used primers are listed in Additional file 1: Table S1. UPM, GSP1, GSP2, and NGSP were primers used for 5′-RACE PCR. CsMRP4-I-F/R and CsMRP4-II-F/R primers were designed for amplification of CsMRP4-I and II fragments through DNA-walking. SF1, SR1, SF2, SR2, and F3 were used for sequencing. b Amplification of the missing 5′-end using RACE-PCR. c RACE-PCR products were confirmed using nested-PCR with UPM and NGSP primers. d The putative *CsMRP4* (GenBank ID: GAA49862.1) was confirmed by DNA-walking. (TIFF 3873 kb)

Additional file 3: Figure S2. The full cDNA coding sequence and deduced polypeptide sequence of CsMRP4. Through 5′-RACE and DNA-walking, the whole cDNA of 4410 bp was verified to encode a polypeptide of 1496 aa. (TIFF 5203 kb)

Additional file 4: Table S2. Identities calculated between CsMRP4 and MRP/SUR/CFTR subfamily members. (PDF 29 kb)

Additional file 5: Figure S3. The residue-by-residue stereochemical quality of the CsMRP4 3D model. Ramachandran plot showed the residues in the most favored regions (90.7%), additional allowed regions (8.4%), generously allowed regions (0.6%), and disallowed regions (0.3%). *Red* (A, B, L), *yellow* (a, b, l, p), and *light yellow* (~a, ~b, ~l, ~p) indicate the most favored regions, allowed regions, and generously allowed regions, respectively. *White* indicates disallowed regions. All the non-glycine and non-proline residues are shown as closed *black squares*, while glycines (non-end) are shown as closed *black triangles*. Disallowed residues are colored in *red*. (TIFF 993 kb)

Additional file 6: Figure S4. Accuracy of the non-bonded atomic contacts of the CsMRP4 3D model. The ERRAT plot shows the overall quality factor of 98.45%. (TIFF 1786 kb)

Additional file 7: CsMRP4.pdb. The model of CsMRP4 was built using YASARA. (PDB 1780 kb)

Additional file 8: Figure S5. Amplification of CsMRP4-NBD1 (a) and purification of the recombinant protein (b). *Abbreviations*: M, molecular marker (kDa); U, uninduced total lysate; I, induced total lysate; S, urea-treated clear supernatant; PT, Ni-NTA pass-through fraction; W, last washing; Elute 1–6, 1st to 6th fraction eluted from an Ni-NTA column. (TIFF 2938 kb)

## Abbreviations

5′-RACE: 5′-rapid amplification of the cDNA ends
aa: Amino acid
ABC: ATP-binding cassette
BSEP: Bile salts export pump
CA: Cholic acid
CDCA: Chenodeoxycholic acid
CFTR: Cystic fibrosis transmembrane conductance regulator
CsMRP4: Multidrug resistance-associated protein 4
DCA: Deoxycholic acid
GCA: Glycocholic acid
LCA: Lithocholic acid
MSD: Membrane-spanning domain
NBD: Nucleotide-binding domain
P-gp: P-glycoprotein
Q-rt-PCR: Quantitative real-time PCR
SUR: Sulfonylurea receptor
TCA: Taurocholic acid
TCDCA: Taurochenodeoxycholic acid
TDCA: Taurodeoxycholic acid
TLCA: Taurolithocholic acid
