# Supplementary material for: Multidrug resistance-associated protein 4 is a bile transporter of Clonorchis sinensis simulated by in silico docking
Source: Parasit Vectors. 2017 Nov 21;10:578. doi: 10.1186/s13071-017-2523-8 (PMC5697364; doi:10.1186/s13071-017-2523-8)
Supplement: Supplementary file 1 — Primer sets used to amplify CsMRP4 cDNA fragments by PCR. (PDF 20 kb) [file 13071_2017_2523_MOESM1_ESM.pdf]

Table S1. Primer sets used to amplify CsMRP4 cDNA fragments by PCR.

Purpose	Targeted region	Forward/ Reverse	Primer sequence (5'→3')
Sequence cloning	CsMRP4-I	F	ATGTTCCTTCATTACGCATGGGT
		R	CCATACTCCACACCATCTGATTTGA
	CsMRP4-II	F	GTGGCAAACTGGCACTATCAGCT
		R	TCATAAATTTGAAAGCACGAAGCGAGTC
Expression cloning	NBD1	F	GGATCCAATGTCTCGGCACGCTGGTTTG
		R	GCATCAAGCTTCCTTCCCTCCTCCATAATAATGA
Q-rt-PCR		F	GAGCTACTGGCTGCTGATTCC
		R	GGGACTCCTTGCCACAGATTC
