# Supplementary figures and images for: Multidrug resistance-associated protein 4 is a bile transporter of Clonorchis sinensis simulated by in silico docking
Source: Parasit Vectors. 2017 Nov 21;10:578. doi: 10.1186/s13071-017-2523-8 (PMC5697364; doi:10.1186/s13071-017-2523-8)

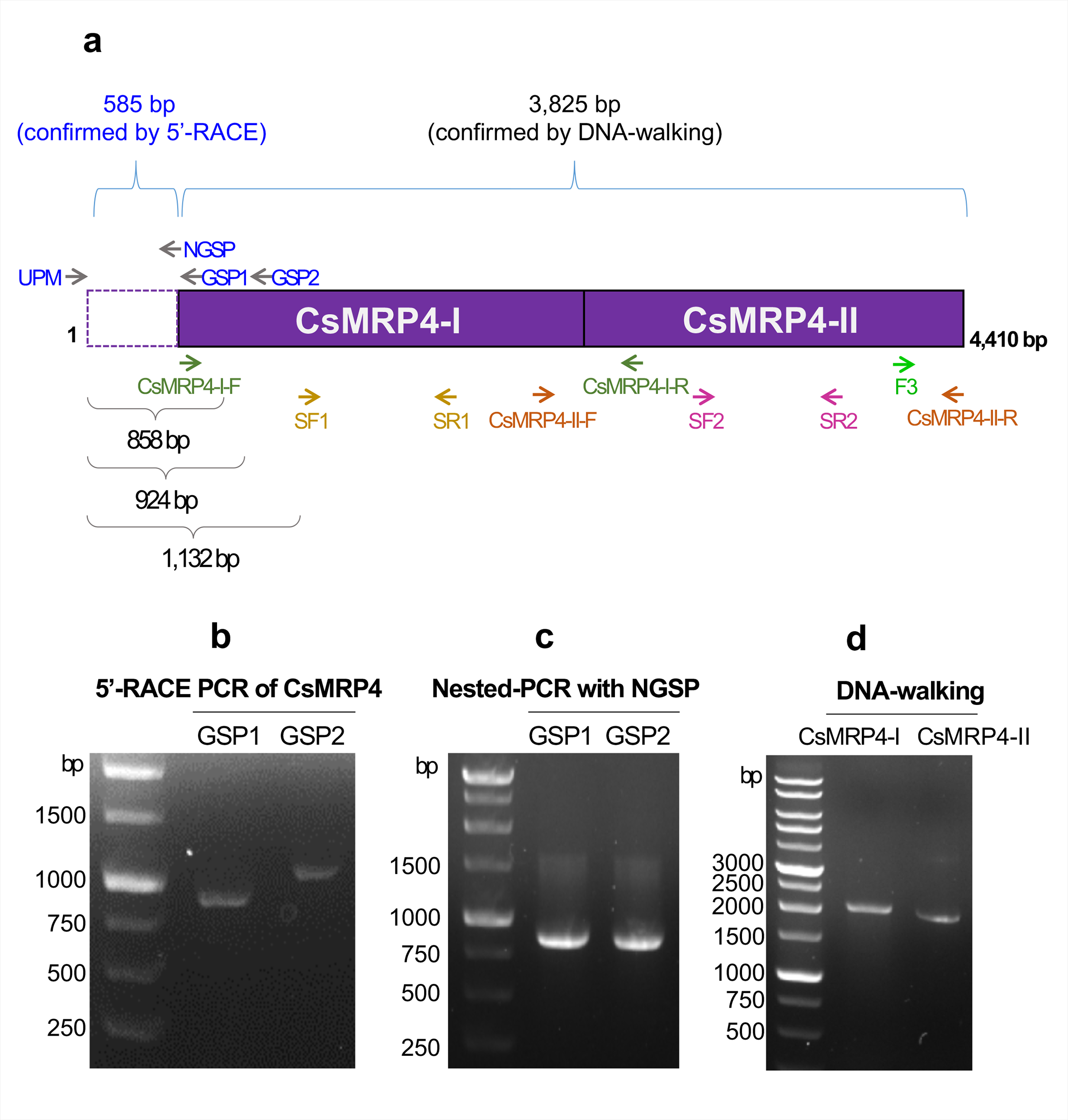

Supplement: Supplementary file 2 — Strategy for obtaining the entire coding cDNA sequence of the CsMRP4 gene. a Whole cDNA (4410 bp) was confirmed by combining 5′-RACE PCR and DNA-walking. The used primers are listed in Additional file 1: Table S1. UPM, GSP1, GSP2, and NGSP were primers used for 5′-RACE PCR. CsMRP4-I-F/R and CsMRP4-II-F/R primers were designed for amplification of CsMRP4-I and II fragments through DNA-walking. SF1, SR1, SF2, SR2, and F3 were used for sequencing. b Amplification of the missing 5′-end using RACE-PCR. c RACE-PCR products were confirmed using nested-PCR with UPM and NGSP primers. d The putative CsMRP4 (GenBank ID: GAA49862.1) was confirmed by DNA-walking. (TIFF 3873 kb) [file 13071_2017_2523_MOESM2_ESM.tif]

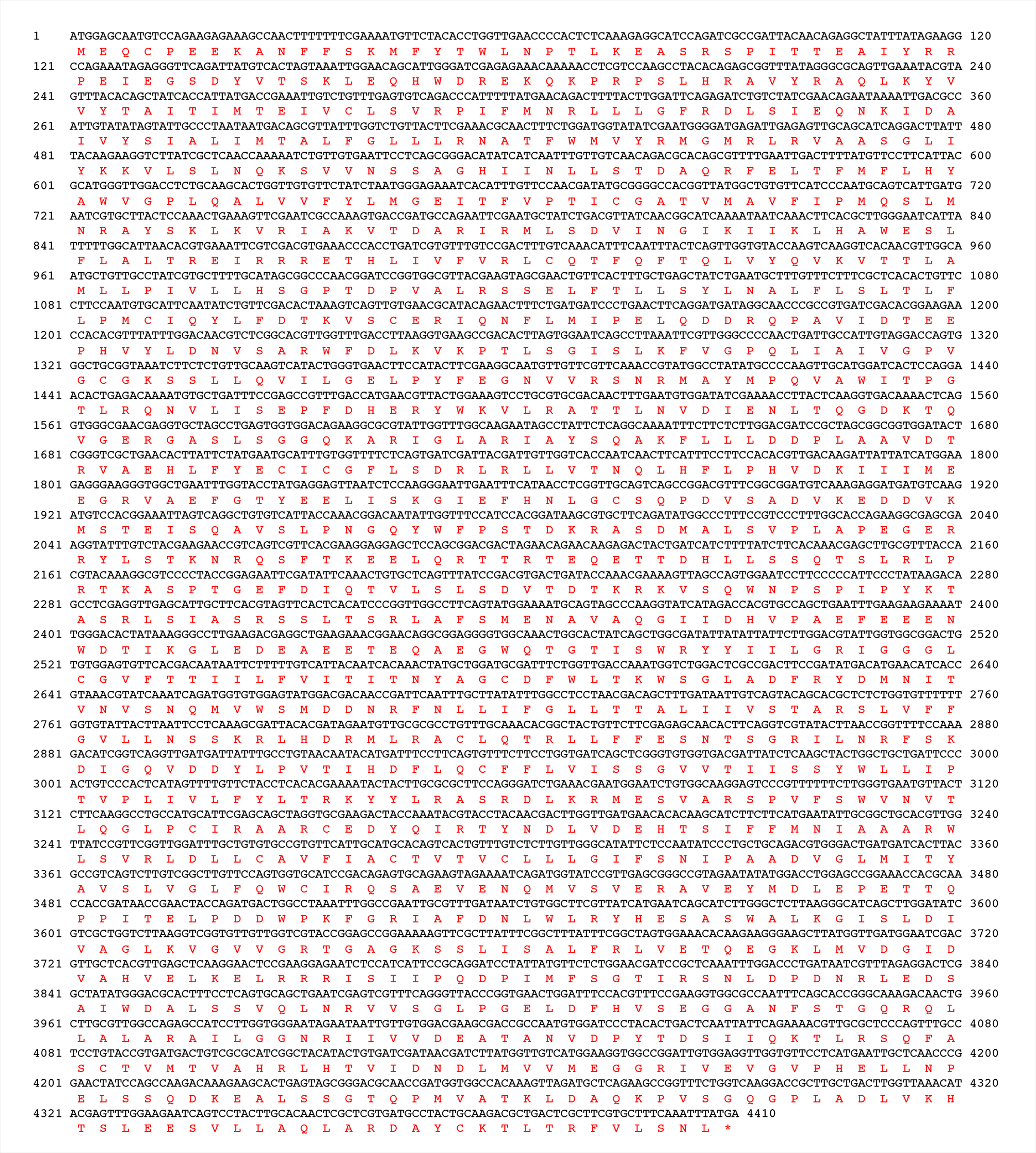

Supplement: Supplementary file 3 — The full cDNA coding sequence and deduced polypeptide sequence of CsMRP4. Through 5′-RACE and DNA-walking, the whole cDNA of 4410 bp was verified to encode a polypeptide of 1496 aa. (TIFF 5203 kb) [file 13071_2017_2523_MOESM3_ESM.tif]

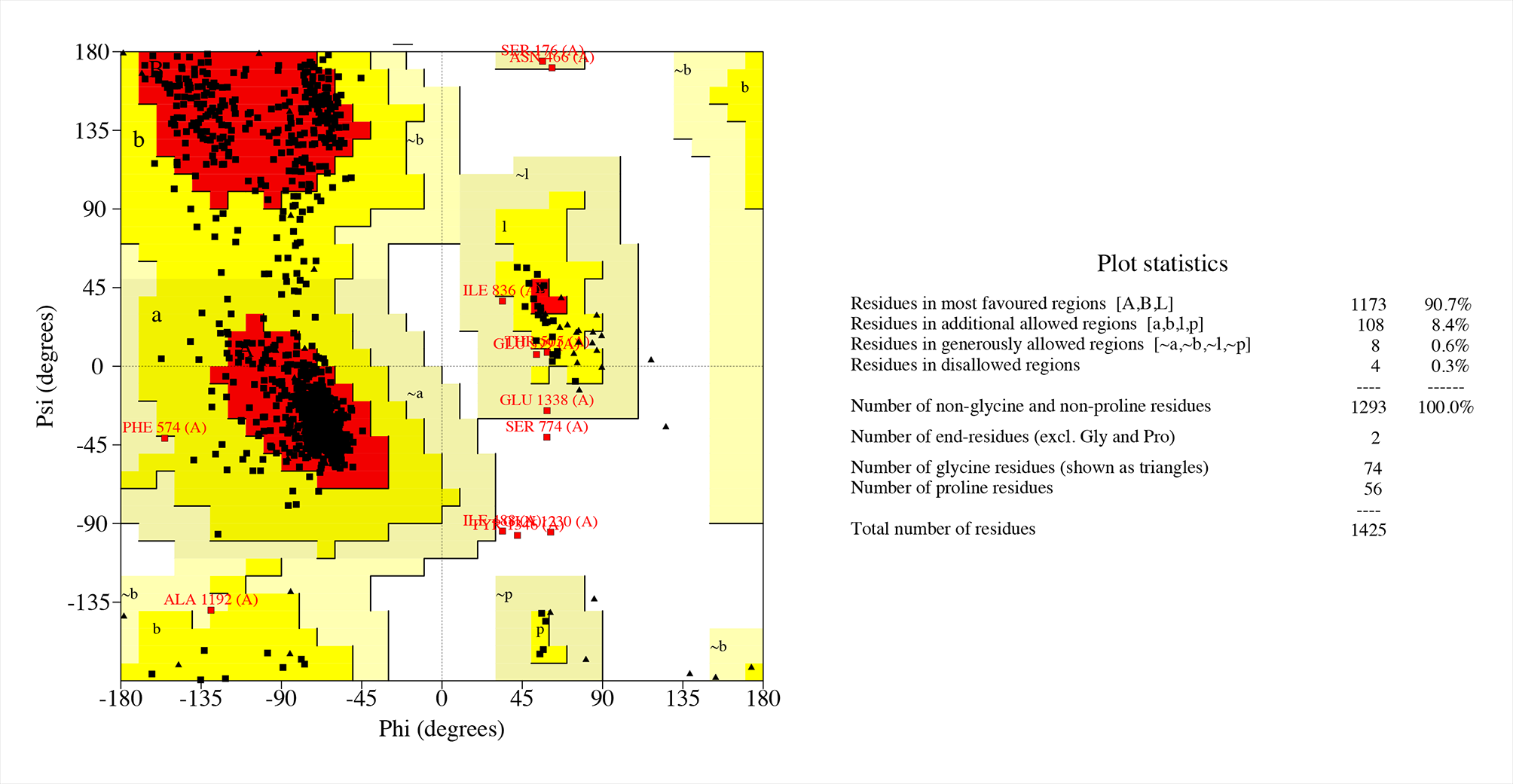

Supplement: Supplementary file 5 — The residue-by-residue stereochemical quality of the CsMRP4 3D model. Ramachandran plot showed the residues in the most favored regions (90.7%), additional allowed regions (8.4%), generously allowed regions (0.6%), and disallowed regions (0.3%). Red (A, B, L), yellow (a, b, l, p), and light yellow (~a, ~b, ~l, ~p) indicate the most favored regions, allowed regions, and generously allowed regions, respectively. White indicates disallowed regions. All the non-glycine and non-proline residues are shown as closed black squares, while glycines (non-end) are shown as closed black triangles. Disallowed residues are colored in red. (TIFF 993 kb) [file 13071_2017_2523_MOESM5_ESM.tif]

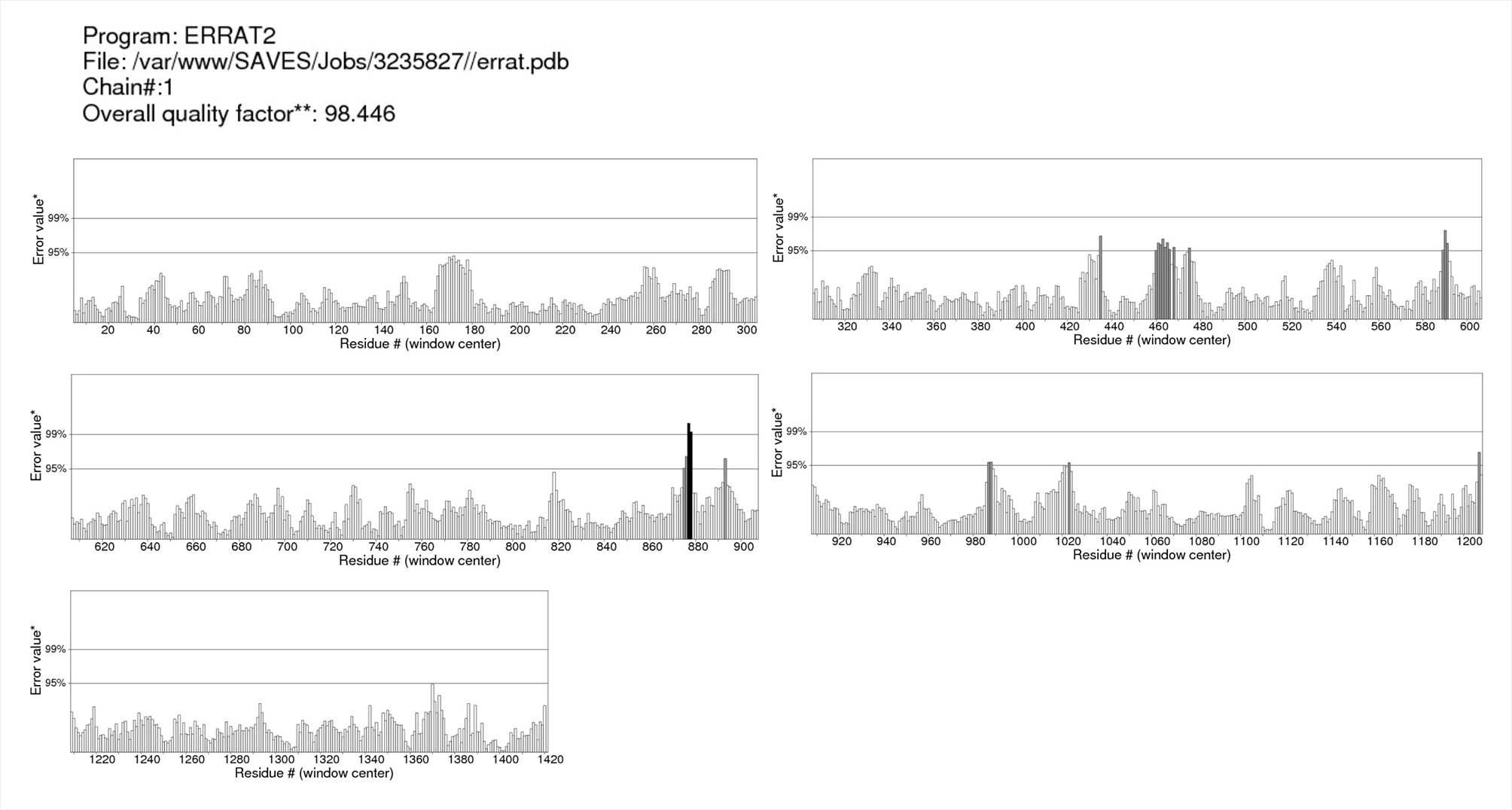

Supplement: Supplementary file 6 — Accuracy of the non-bonded atomic contacts of the CsMRP4 3D model. The ERRAT plot shows the overall quality factor of 98.45%. (TIFF 1786 kb) [file 13071_2017_2523_MOESM6_ESM.tif]

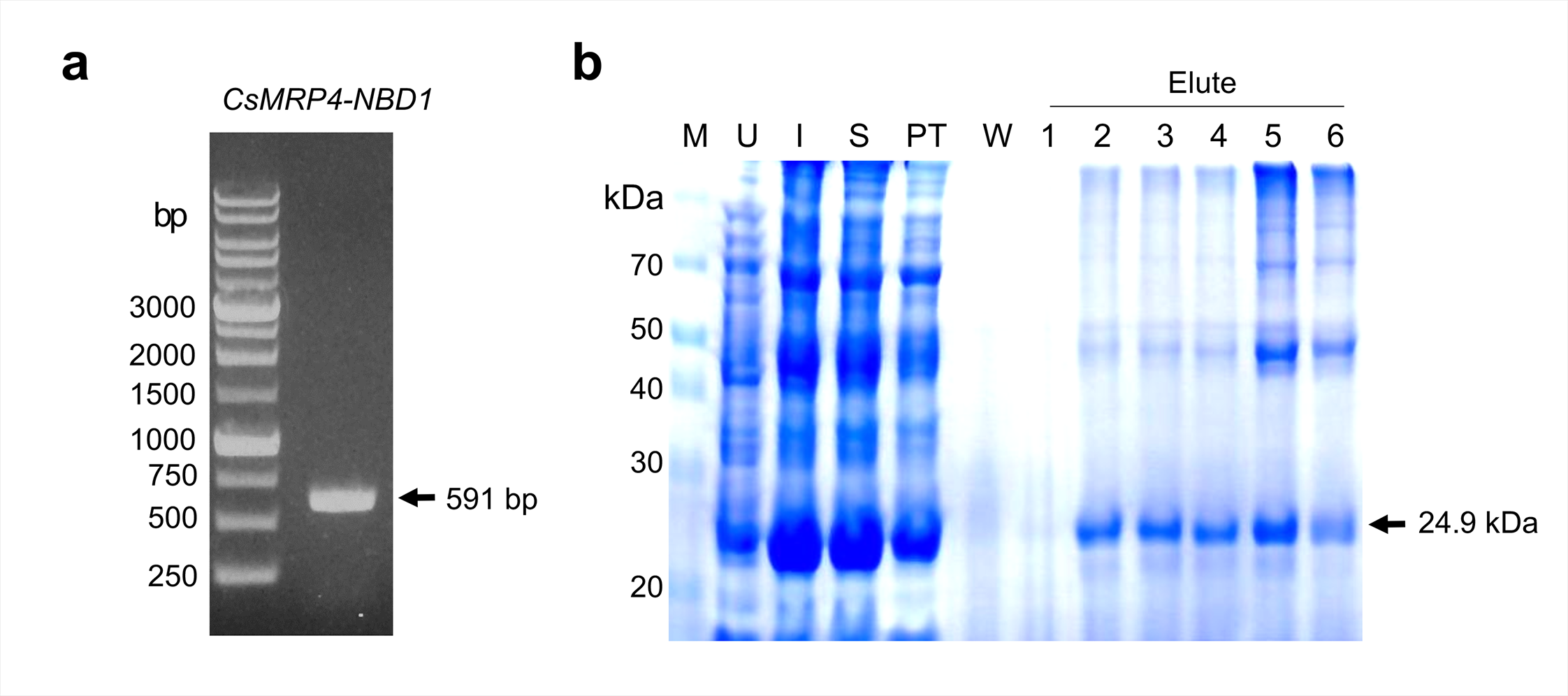

Supplement: Supplementary file 8 — Amplification of CsMRP4-NBD1 (a) and purification of the recombinant protein (b). Abbreviations: M, molecular marker (kDa); U, uninduced total lysate; I, induced total lysate; S, urea-treated clear supernatant; PT, Ni-NTA pass-through fraction; W, last washing; Elute 1–6, 1st to 6th fraction eluted from an Ni-NTA column. (TIFF 2938 kb) [file 13071_2017_2523_MOESM8_ESM.tif]
