# Supplementary material for: Multidrug resistance-associated protein 4 is a bile transporter of Clonorchis sinensis simulated by in silico docking
Source: Parasit Vectors. 2017 Nov 21;10:578. doi: 10.1186/s13071-017-2523-8 (PMC5697364; doi:10.1186/s13071-017-2523-8)
Supplement: Supplementary file 4 — Identities calculated between CsMRP4 and MRP/SUR/CFTR subfamily members. (PDF 29 kb) [file 13071_2017_2523_MOESM4_ESM.pdf]

Table S2. Identities calculated between CsMRP4 and MRP/SUR/CFTR subfamily members.

	CsMRP4	HsMRP4	HsMRP5	HsMRP8	HsMRP9	HsMRP1	HsMRP3	HsMRP2	HsMRP6	HsCFTR	HsSUR1	HsSUR2	CsMRP7	HsMRP7
CsMRP4	-													
HsMRP4	**39.0**	-												
HsMRP5	31.8	35.8	-											
HsMRP8	30.1	34.0	40.5	-										
HsMRP9	31.0	35.4	45.6	49.0	-									
HsMRP1	33.2	37.7	35.6	31.8	34.2	-								
HsMRP3	31.6	35.8	33.7	33.5	32.7	57.9	-							
HsMRP2	31.2	37.6	35.5	31.1	32.7	49.0	47.9	-						
HsMRP6	31.2	33.6	31.9	30.5	31.4	45.3	43.5	38.8	-					
HsCFTR	29.0	35.2	26.4	28.1	26.8	27.4	27.0	27.5	26.7	-				
HsSUR1	30.5	32.6	31.2	28.4	29.6	33.7	32.9	31.7	31.1	25.5	-			
HsSUR2	30.2	32.5	31.9	29.3	28.7	33.6	33.5	32.0	30.8	26.5	68.9	-		
CsMRP7	29.6	31.4	27.3	26.0	28.2	30.6	30.5	29.3	29.7	25.2	26.7	26.9	-	
HsMRP7	31.7	35.1	32.8	30.6	31.9	33.2	34.5	32.9	31.8	27.3	30.2	30.2	38.4	-
